# The impact of protein S and tissue factor pathway inhibitor on coagulation, assessed with thrombin generation, in women starting combined oral contraceptives

**DOI:** 10.1016/j.rpth.2025.102981

**Published:** 2025-07-25

**Authors:** Jesper Strandberg, Jette Nybo, Inger Lise Gade, Søren Risom Kristensen

**Affiliations:** 1 The Coagulation Unit, Department of Clinical Biochemistry, Aalborg University Hospital, Aalborg, Denmark; 2 Department of Clinical Medicine, Aalborg University, Aalborg, Denmark; 3 Department of Haematology and Clinical Cancer Research Centre, Aalborg University Hospital, Aalborg, Denmark

**Keywords:** APC, aPS, aTFPI, cofactor, combined oral contraceptives, protein S, thrombin generation

## Abstract

**Background:**

Combined oral contraceptives (COCs) are associated with an increased risk of venous thromboembolism. Several changes are induced in both coagulant and anticoagulant factors, of which the impact on protein S (PS) and tissue factor pathway inhibitor (TFPI) may be especially important. The global thrombin generation (TG) assay, which accounts for all pro- and anticoagulant factors, can be used to evaluate the effect of the changes of PS and TFPI using antibodies to eliminate the effect of these inhibitors.

**Objectives:**

The aim of this study was to investigate the effect of PS, TFPI, and activated protein C (APC) on coagulation, as assessed with the TG assay in individuals before and after starting COCs.

**Methods:**

Twenty-four women between 15 and 34 years of age who were starting COC treatment were included in the study. Blood samples were drawn at baseline, before first COC dose, and at follow-up, approximately 3 to 4 months later. TG assays were performed on all samples, with the addition of anti-PS, anti-TFPI, and APC, to evaluate their impact on TG.

**Results:**

A reduction in APC sensitivity was demonstrated after COC start, reflected in a twofold increased normalized APC sensitivity ratio. TG potential increased significantly after addition of anti-PS and anti-TFPI, both at baseline and after 3 months of COC treatment, but increased relatively less at follow-up.

**Conclusion:**

While we previously found only modest COC-induced decreases in PS activity and TFPI levels in this population, indicated a substantial reduction of anticoagulant activity after 3 months of COC use.

## Essentials

• COCs are associated with procoagulant alterations.
• TG assay can be used to assess overall thrombogenicity.
• TG was assessed using antibodies against protein S and tissue factor pathway inhibitor.
• Results indicated a substantial reduction in anticoagulant activity after 3 months of COC use.

## 1 Introduction

Combined oral contraceptives (COCs) are a widely used method of contraception, with an estimated 150 million users worldwide [1,2]. COCs contain a synthetic estrogen, ethinylestradiol (EE), which is associated with increased risk of venous thromboembolism (VTE), and a progestogen [[2], [3], [4], [5], [6], [7], [8]]. COCs are generally classified into 4 generations based on the type of progestogen or based on their content [2,9,10]. Second generation COCs combine 20 to 30 μg EE with 100 to 150 μg levonorgestrel, such that the higher levonorgestrel dose helps counteract the estrogenic effect of 30 μg EE. Third generation COCs also contain 20 to 30 μg EE, but it is combined with different progestogens, such as desogestrel or gestodene, which have weaker antiestrogenic effects, resulting in a greater impact on the coagulation system and an increased risk of VTE compared with second generation COCs [9,11,12]. Fourth generation COCs contain the progestogen drospirenone and have similar effects as third generation COCs. Some COC formulations remain unclassified. A newer COC, containing the natural estrogen estetrol, has been introduced, and may reduce the risk of VTE due to its lower impact on coagulation [11,[13], [14], [15], [16], [17]].

COC users have a 2- to 6-fold higher risk of VTE than nonusers, and the risk is higher for third generation COCs than second generation COCs [[2], [3], [4], [5], [6], [7], [8]]. Estrogen leads to higher levels of certain procoagulant factors and reduced levels of anticoagulant factors [2,[18], [19], [20]]. While most of these changes are relatively modest when measured in plasma, the combined effect can be investigated using the global thrombin generation (TG) assay, which includes all procoagulant and anticoagulant factors, and can reveal a state of hypercoagulability in COC users [[21], [22], [23], [24], [25], [26], [27]].

Reduced plasma levels of the natural anticoagulants protein S (PS) and tissue factor pathway inhibitor (TFPI) under the influence of COCs have been described previously [[18], [19], [20]], and this has been suggested to be a significant cause of the increased thrombogenicity associated with COCs [22,28,29]. PS is crucial for maintaining hemostatic balance, and deficiencies or dysfunctions in PS are linked to an increased risk of VTE [[30], [31], [32]]. PS serves as a nonenzymatic cofactor to activated protein C (APC), which inactivates coagulation factors (F)Va and FVIIIa, and also as a cofactor to TFPI-inactivating FXa. Furthermore, PS may interact directly to inhibit procoagulant factors, such as FXa and coagulation FIXa [30,[32], [33], [34], [35], [36]].

TFPI is a key regulatory protein in the coagulation cascade by preventing excessive TG [[37], [38], [39], [40]]. The primary function of TFPI is to inhibit the initiation of coagulation, triggered by tissue factor (TF) exposure. TFPI exerts its anticoagulant function in a 2-step inhibition process. First, TFPI binds to and inhibits FXa, forming a complex. Second, the TFPI:FXa complex inhibits the TF:FVIIa complex, thus terminating TF:FVIIa-catalyzed FX activation and preventing further activation of FX and FIX [[37], [38], [39], [40]]. PS also stabilizes the TFPI:FXa complex, allowing more effective inhibition of the TF:FVIIa complex and thereby reducing TG [35,[37], [38], [39], [40]].

The effects of PS and TFPI on TG have been thoroughly studied, both with and without APC. It has been shown that TG increases in normal plasma when either PS or TFPI are absent (determined using antibodies against each of the anticoagulants), with a more pronounced effect observed when TFPI was missing compared to PS. Adding APC reduced TG in both situations [37,38,[40], [41], [42], [43], [44], [45], [46], [47]]. In plasma lacking PS, inhibiting TFPI activity led to a significant further increase in TG [43], whereas in plasma lacking TFPI, inhibiting PS did not cause a noticeable additional increase. TG in women taking COCs has been evaluated previously, showing higher TG in women receiving COCs [23,25,[48], [49], [50], [51], [52]]. Evaluation of TG in women starting COCs has, to our knowledge, only been sparsely investigated and only in small cohorts [24,50,53], including one of our previous studies [54], in which we also measured the concentrations of PS and TFPI, showing minor decreases after initiation of COCs. To our knowledge, evaluation of TG in women starting COCs by assessing the impact of PS and TFPI using antibodies to eliminate the effects of the inhibitors has not been performed before.

The aim of this study was to investigate the effect of PS and TFPI on the coagulation system, assessed with TG assay, before and after starting treatment with COCs.

## 2 Methods

### 2.1 Study design and study population

The study population and inclusion and exclusion criteria have been previously described [54]. In short, 26 healthy women who were about to start treatment with COCs were included. Participants completed a questionnaire and were requested to provide a baseline blood sample before commencing COC treatment, followed by a follow-up blood sample approximately 3 menstrual cycles after initiation of treatment. The primary COC choice recommended in Denmark is second generation.

The study was conducted in accordance with the Declaration of Helsinki and received approval from the North Denmark Region Committee on Health Research Ethics (N-20200098). Informed written consent was obtained from all participants, with additional consent required from both parents for those aged <18 years.

### 2.2 Materials

Corn trypsin inhibitor and antibodies against TFPI (aTFPI) were acquired from Haematologic Technologies, Prolytix; polyclonal antibodies against PS (aPS) from DAKO, Agilent; and APC from Enzyme Research Laboratories. The trigger platelet poor plasma (PPP)-Reagent Low, FluCa buffer, CaCl_2_, and thrombin calibrator were all acquired from Diagnostica Stago S.A.S.

### 2.3 Blood collection

Venous blood was collected from the antecubital fossa using a 21-gauge needle into 3.2% (w/v) trisodium citrate Monovette tubes (Sarstedt) for the TG analyzes. The first 1 to 2 mL of blood was discarded. The Monovette samples were centrifuged twice at 2500 × *g* for 15 minutes at 20 °C immediately after collection in accordance with current guidelines [55]. Plasma samples were stored at −80 °C until analysis.

### 2.4 Biochemical investigation

TG was determined on Fluoroskan Ascent (Thermo Scientific) using calibrated automated thrombography, as described by Kristensen et al. [48]. TG was measured following the manufacturer’s protocol (80 μL plasma combined with 20 μL of trigger solution labeled PPP-Reagent Low). We used PPP-Reagent Low (1 pM TF) to enhance sensitivity to endogenous variations in TG [56]. PPP-Reagent Low has also been used in previous studies with antibodies to demonstrate the effects of PS and TFPI [43,57]. The impact of PS and TFPI on TG was assessed using aPS and aTFPI. The aTFPI concentration was the same as used in a previous study [58]. Alshaikh [24] used a cocktail of 4 antibodies, which resulted in full inhibition. As each antibody achieves approximately 90% to 95% inhibition individually (Tilman Hackeng, personal communication), addition of another antibody (Thermo Fisher, Invitrogen) did not produce a detectable effect. Therefore, we proceeded using only the latter in our experiments.

For PS inhibition, we tested various antibodies with different effects on TG, but ultimately chose antibodies from DAKO, which were the same type as those employed in studies by Tchaikovski et al. [57] and produced a consistent inhibitory response. Different concentrations were tested, and the concentration of 2.8 μM was found optimal, in accordance with Tchaikovski et al. [55].

To evaluate the effect of APC, we calculated the APC sensitivity ratio, normalized to normal plasma. The assay was performed according to standard procedure [23] in the presence and absence of APC. The endogenous thrombin potential (ETP) was determined, and a total normalized APC sensitivity ratio (nAPCsr) was calculated:

$$nAPCsr=\left(\frac{ETP\left(+APC\right)}{ETP\left(-APC\right)}\right)/\left(\frac{ETP\left(NP\right)\;\left(+APC\right)}{(ETP\left(NP\right)\left(-APC\right)}\right)$$

where ETP(NP) represents the ETP values obtained from pooled normal plasma.

We investigated TG in the samples at baseline and follow-up of plasma without additions (control) and plasma after addition of aPS, aTFPI, APC, or aTFPI in combination with APC.

Plasma was incubated with corn trypsin inhibitor at a concentration of 40 μg/mL in plasma to inhibit contact system activation, and either physiological saline (control), 2.8 μM of polyclonal aPS, 100 μg/mL of aTFPI, 1.0 nM of APC, or a combination of aTFPI and APC was added (final concentrations in plasma for all additions were made in equal volumes, ie, same small dilution of plasma). APC concentration was adjusted to achieve 10% of the ETP in normal plasma. After the additions of aPS and aTFPI, the samples underwent incubation for 15 minutes at 37 °C before 80 μL of the mixtures (same amount of plasma in each) was used for TG. APC was not preincubated but was added directly before TG.

Coagulation, and thus TG, was initiated by adding 20 μL of the trigger reagent PPP-Reagent Low (1 pM TF, 4 μM phospholipids), and 20 μL FluCa buffer containing a fluorescent substrate (Z-Gly-Gly-Arg-AMC) and CaCl_2_. The fluorescent substrate is used to measure TG indirectly through fluorescence. To account for variations in plasma color, each plasma measurement was calibrated against the same plasma mixed with 20 μL thrombin calibrator, performed in duplicate. Thrombinoscope software (Thrombinoscope BV) was used to calculate Lag time, ETP, peak height values (Peak), and time-to-peak (ttPeak).

Normal plasma was prepared locally by pooling plasma from 8 healthy volunteers collected in 3.2% (w/v) trisodium citrate Monovette tubes (Sarstedt) and centrifuged twice at 2500 × *g* for 15 minutes at 20 °C. The group of healthy volunteers consisted of both males and females. None of the female participants were using COCs at the time of sampling, and the plasma had no APC resistance.

### 2.5 Statistical considerations

Data distributions were assessed by histograms, and quantile–quantile plots and were found to be normally distributed. All differences between baseline and follow-up results were parametrically tested using paired Student’s *t*-test and were considered significant when the *P* value was < .05. Absolute and relative individual differences were calculated. Results are presented as mean values ± SD.

## 3 Results

### 3.1 Basic characteristics

The study population was described in a previous article [54]. Twenty-six subjects were included in the study, with a median age of 17 years at the day of the first blood sample. Six of the subjects had used COCs previously but not within the 3 months prior to inclusion. Twenty-four subjects (92%) received second generation COCs, 4 of whom received a mixture of 20 μg EE and 100 μg levonorgestrel, and the remaining 20 received 30 μg EE and 150 μg levonorgestrel. Two subjects did not receive second generation COCs and were therefore excluded. The characteristics of the study population are presented in Table 1.

**Table 1:** Basic characteristics of the study population^a^ (*N* = 24).

**Age (y), median (range)**	17 (15-34)
**Ethinylestradiol dose (20 μg/30 μg)**	4/20
**COC generation, second**	24

COC, combined oral contraceptive.

a All study participants were Caucasian.

The APC resistance was estimated by calculation of the mean nAPCsr for the entire group and stratified according to EE dose. Results are presented in Table 2. For the entire cohort (*N* = 23), the nAPCsr increased significantly from 1.05 at baseline to 2.14 after 3 months, indicating an increased TG in the presence of APC, thus showing an increased APC resistance under the influence of COC. The group using 20 μg ethinylestradiol (*n* = 4) had essentially the same nAPCsr as the group receiving 30 μg ethinylestradiol.

**Table 2:** Normalized APC sensitivity ratio.

	Baseline Mean ± SD	Follow-up Mean ± SD	*P*
**All subjects (*N* = 23)**^a^	1.05 ± 0.39	2.14 ± 0.62	> .0001
**20 μg ethinylestradiol (*n* = 4)**	0.96 ± 0.57	1.98 ± 0.16	.06
**30 μg ethinylestradiol (*n* = 19)**	1.06 ± 0.36	2.17 ± 0.68	> .0001

APC, activated protein C; nAPCsr, normalized APC sensitivity ratio.

a Missing baseline results for APC for 1 subject.

### 3.2 TG measurements

**Figure 1:**
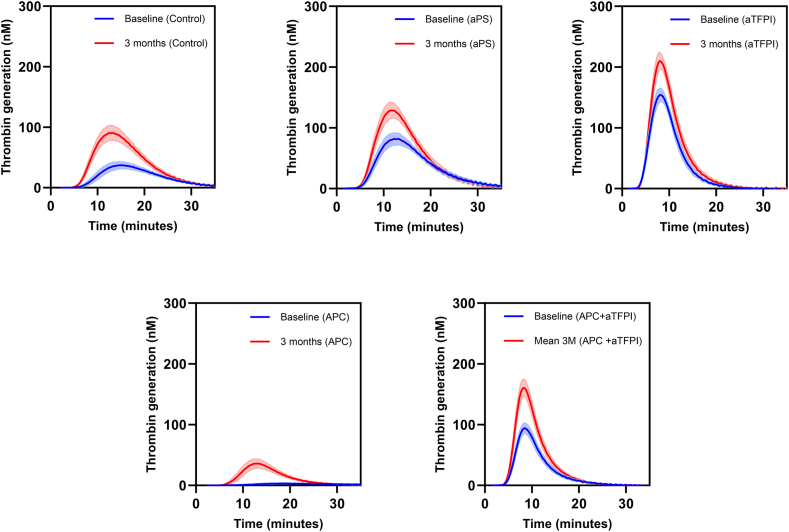
Thrombin generation assay results at baseline and at follow-up. Each curve shows the mean of the participants and 95% CI at baseline (blue curve) and at 3 months follow-up (red curve). APC, activated protein C; aPS, anti-PS; aTFPI, antitissue factor pathway inhibitor.

We assessed TG in patient plasma without the addition of any other substances (control), as well as after the addition of aPS, aTFPI, APC, and APC in combination with aTFPI. Baseline samples and 3-month follow-up samples were analyzed, and the results are presented in Table 3 and visualized in Figure 1. Relative individual differences (RIDs) were defined as

$$\left(\;\text{Result}s_{\text{follow}-\text{up}}\;-\;\text{Result}s_{\text{baseline}}\right)/\text{Result}s_{\text{baseline}}\times 100\%$$

**Table 3:** Thrombin generation results, mean values ± SD (*N* = 24).

Parameter	Baseline	Follow-up	Absolute individual difference	Relative individual difference (%)	*P*
**Control**					
Lag time, min	8.2 ± 1.4	6.7 ± 0.6	−1.5 ± 1.3	−17 ± 12	.260
Peak, nmol/L	38 ± 16	91 ± 30	53 ± 24	155 ± 83	< .0001
ETP, nmol/L·min	567 ± 187	1183 ± 302	617 ± 246	121 ± 64	< .0001
ttPeak, min	15.1 ± 1.4	13.2 ± 0.8	−1.9 ± 1.3	−12 ± 8	< .0001
**aPS**					
Lag time, min	6.3 ± 0.8	6.0 ± 0.6	−0.24 ± 0.9	−3 ± 14	.182
Peak, nmol/L	84 ± 26	132 ± 32	48 ± 24	63 ± 35	< .0001
ETP, nmol/L·min	1121 ± 143	1508 ± 209	387 ± 137	35 ± 12	< .0001
ttPeak, min	13.1 ± 1.3	12.2 ± 1.0	−1.0 ± 1.2	−7 ± 9	.0004
**aTFPI**					
Lag time, min	4.3 ± 0.4	4.5 ± 0.3	0.2 ± 0.4	5 ± 9	.08
Peak, nmol/L	159 ± 28	218 ± 34	59 ± 22	39 ± 17	< .0001
ETP, nmol/L·min	1094 ± 146	1483 ± 222	379 ± 126	35 ± 11	< .0001
ttPeak, min	8.3 ± 0.7	8.3 ± 0.6	−0.1 ± 0.4	−0.5 ± 5.5	.501
**APC**^a^					
Lag time, min	11.8 ± 5.5	7.7 ± 0.8	−4.4 ± 4.8	−29 ± 23	.001
Peak, nmol/L	4 ± 2	38 ± 20	33 ± 20	980 ± 657	< .0001
ETP, nmol/L·min	83 ± 41	380 ± 181	290 ± 172	405 ± 262	< .0001
ttPeak, min	24.6 ± 11.4	13.4 ± 2.0	−11.8 ± 9.9	−42 ± 18	< .0001
**APC + aTFPI**					
Lag time, min	5.1 ± 0.5	5.1 ± 0.4	−0.01 ± 0.4	0.2 ± 8.3	.872
Peak, nmol/L	97 ± 22	165 ± 36	69 ± 25	74 ± 29	< .0001
ETP, nmol/L∗min	669 ± 169	1064 ± 210	395 ± 120	63 ± 25	< .0001
ttPeak, min	8.4 ± 0.6	8.3 ± 0.5	−0.2 ± 0.5	−2 ± 6	.101

APC, activated protein C; aPS, anti-protein S; aTFPI, antitissue factor pathway inhibitor; ETP, endogenous thrombin potential; ttPeak, time-to-peak.

a *N* = 23 (no baseline results for 1 subject).

**Figure 2:**
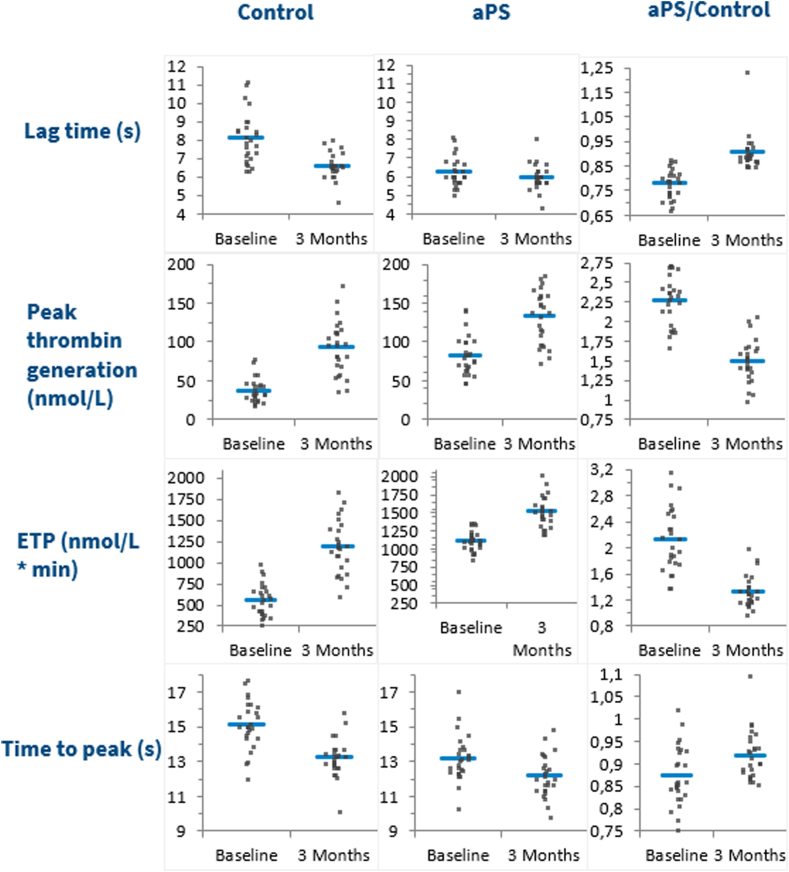
Results of thrombin generation assay for control situation and with aPS added in first and second columns; ratios are depicted in the third column. aPS, anti-PS; ETP, endogenous thrombin potential.

**Figure 3:**
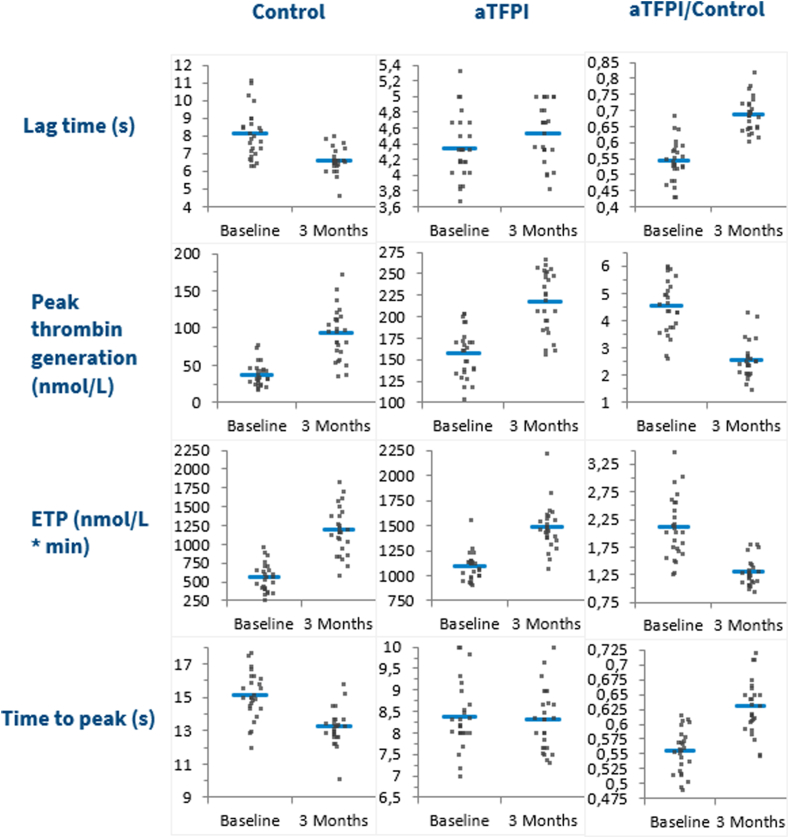
Results of thrombin generation assay for control situation and with aTFPI added in first and second columns; ratios are depicted in the third column. aTFPI, antitissue factor pathway inhibitor; ETP, endogenous thrombin potential.

**Figure 4:**
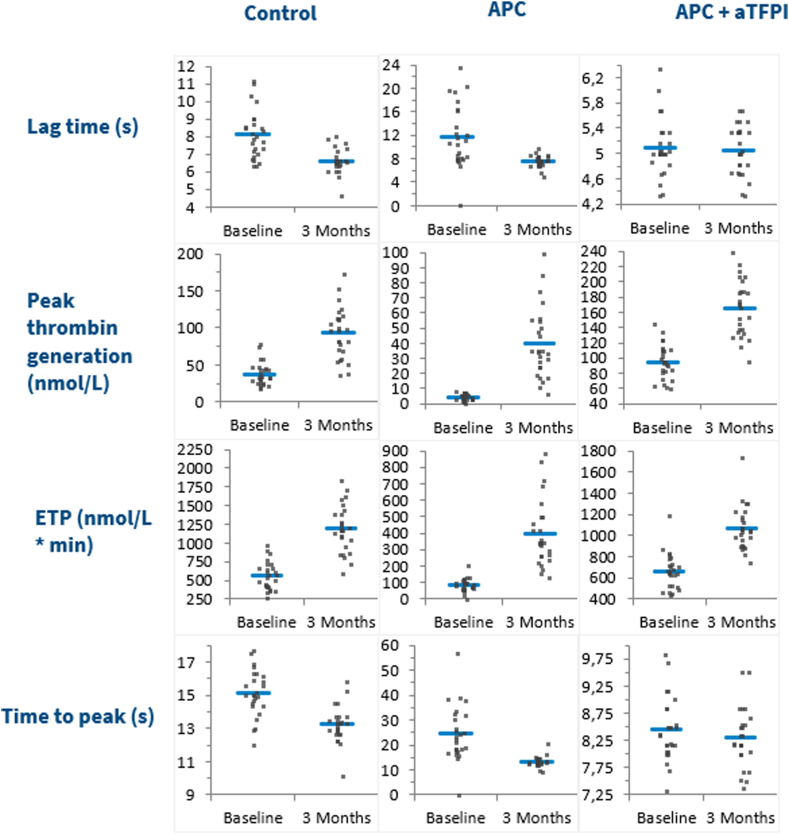
Results of thrombin generation assay for control, APC, and APC+aTFPI. APC, activated protein C; aTFPI, antitissue factor pathway inhibitor; ETP, endogenous thrombin potential.

When we compared the effect of the additions, we first compared RIDs after each of the additions with RID of the control (ie, using the results in Table 3). Subsequently, we compared the ratio for each individual between the results after each of the additions at baseline and at 3 months follow-up and the control situation at baseline and at 3 months follow-up, respectively (shown in Figure 2, Figure 3, Figure 4).

### 3.3 Control plasma

TG at the follow-up was substantially increased by COCs, especially Peak (+155%) and ETP (+121%). A small decrease of ttPeak (−12%) was also observed.

### 3.4 aPS treatment

Addition of aPS removed the anticoagulation activity of PS. This increased TG at baseline as well as at follow-up. Peak values increased 63% and ETP 35%, whereas ttPeak was reduced by 7% at follow-up compared with baseline (Table 3). However, these changes after the start of COCs were much smaller than in the controls (+155%, +121%, and −12%, respectively [Table 3]). Figure 2 shows the values for each individual in the control situation and after addition of aPS as well as the ratio between these values at baseline and at the follow-up. At baseline, ratios were 2.28 for Peak and 2.12 for ETP, whereas at the follow-up, ratios are only 1.51 and 1.32, respectively. For ttPeak, the ratios also showed a slightly smaller reduction at follow-up of 0.92 (compared with 0.87).

Thus, the addition of aPS has a relatively smaller effect on the samples at follow-up, indicating a substantial effect of the reduction of PS caused by COCs.

### 3.5 aTFPI treatment

Addition of aTFPI removed the anticoagulation activity of TFPI. This also increased TG at baseline as well as at follow-up. Peak values increased +39% and ETP +35%, whereas Lag time and ttPeak were unchanged at follow-up compared with baseline (Table 3). This increase after the start of COCs was much smaller than for the controls (Table 3). Figure 3 shows that the ratios between the activities after the addition of aTFPI and the activities in the control situation were much more pronounced in the baseline samples than at the follow-up: at baseline these ratios were 4.51 for Peak and 2.08 for ETP, whereas at follow-up, these ratios are only 2.57 and 1.29, respectively. For ttPeak, the ratios also exhibited a slightly smaller reduction at follow-up of 0.62 compared with 0.55.

Thus, the addition of aTFPI has a smaller effect on the samples at follow-up than at baseline, indicating a substantial effect of the reduction of TFPI caused by COCs. Compared with aPS, the reduction of the effect on Peak value was more marked, whereas the effect on ETP was almost identical.

### 3.6 Treatment with APC + aTFPI

Addition of APC increased the activity of the APC:PS system. TG increased considerably at follow-up compared with baseline (Table 3). Peak values increased 980% and ETP 405% (in accordance with the increased nAPCsr), whereas Lag time and ttPeak were reduced by 29% and 42%, respectively. However, the addition of APC as well as aTFPI resulted in increased APC:PS activity together with removal of the anticoagulation activity of TFPI. This increased TG considerably both at baseline and at follow-up, indicating a substantial effect of TFPI. The effect was also relatively smaller at 3-month follow-up than at baseline (Table 3), especially for Peak and ETP, which are approximately half of the control values. This effect was enhanced when compared at the individual level (Figure 3).

Thus, the addition of aTFPI in combination with APC results in TG activity not much lower than that with aTFPI alone. This illustrates that TFPI has a profound effect on TG. Furthermore, the effect on the samples at follow-up indicates a substantial effect of the reduction of TFPI caused by COCs.

## 4 Discussion

In this study, we investigated the impact of PS, TFPI, and APC on the general coagulation system, assessed using TG assay, after the start of COC treatment. Our overall findings indicate that COC use leads to a significant increase in TG potential and a reduction in APC sensitivity and also indicate profound role of PS and TFPI in the procoagulant changes after initiation of COCs. Whereas our previous study [54] showed only small or discrete changes in levels of PS and TFPI in the same women starting COCs, the results of this study using antibodies to eliminate the effect of the inhibitors demonstrate a substantial increase in TG after 3 months of COC use, indicating that the measured small alterations in coagulant factors may have great impact on overall coagulation, ie, the effects are much more marked than indicated by the measurements of the single factors.

Previous studies have shown that women using COCs have increased TG [23,25,[48], [49], [50], [51], [52]]. However, there has been limited research on TG in women starting COCs [24,50,53,54], and to our knowledge, there has been no research investigating the impact of PS and TFPI on TG using the present approach in women starting COCs.

### 4.1 Impact of APC

When APC is added, it complexes with PS to inactivate FVa and FVIIIa. The concentration of APC (1 nM) was chosen so that ETP was approximately 10% of that in the absence of APC, in accordance with most guidelines for measuring nAPCsr [59,60]. It has previously been shown with this method that it is possible to demonstrate the APC resistance caused by COCs and to discriminate between different generations of COCs, and it may be a feasible method to compare thrombogenicity of newer formulations of COCs [23,[59], [60], [61]]. The observed increase in nAPCsr from baseline to follow-up was expected, and as EE is known to induce a dose-dependent increase in APC resistance, the slightly lower nAPCsr in the 20-μg EE group compared to the 30-μg group aligns with existing literature [23,[59], [60], [61]]. However, the difference is minimal, probably because the higher dosage of EE is balanced by a correspondingly higher dosage of gestagen (100 and 150 μg levonorgestrel correspond to 20 and 30 μg EE, respectively).

We found a doubling of nAPCsr by the second generation COCs used here. Previously, in line with our study, the Oral Contraceptive and Haemostasis Study Group [61], Rosing et al. [59], and Tchaikovski et al. [23] found that nAPCsr increases nearly 2-fold for second generation COCs, and the nAPCsr for third generation COCs is even higher. Douxfils et al. [62] showed a similar trend but higher nAPCsr in general, whereas Morimont et al. [60] found significantly higher nAPCsr for both second (close to 3-fold) and third generation COCs. Different methodologies were used, which limit direct detailed comparison of results, but the results show a similar overall trend. In a Cochrane review from 2014, de Bastos et al. [63] concluded that APC resistance is associated with higher VTE risk due to protein C’s inability to cleave activated coagulation FV and FVIII, thus resulting in a more prothrombotic state.

A higher nAPCsr has been described for FV Leiden-positive individuals [[64], [65], [66], [67]]. The 2 subjects heterozygous for FV Leiden in our study had nAPCsr values below the mean of the general cohort were. Thus, we did not see a synergistic effect of the presence of FV Leiden in these 2 particular individuals, and they did not impact nAPCsr for the group.

### 4.2 Impact of PS inhibition

PS is a cofactor for APC and TFPI. The complex with APC inactivates FVa and FVIIIa, and PS is also a cofactor for TFPIα for inactivating FXa. It has also been reported that PS may have a direct inhibitory effect on FIXa and FXa, although this has not been extensively investigated [30,[32], [33], [34], [35], [36]].

To investigate the effect of PS, we assessed TG after addition of aPS (thereby removing the effect of PS), and we observed significantly higher TG than in the control in which the effect of PS was present, both at baseline and follow-up. The addition of aPS had a considerable effect, showing that PS plays a critical anticoagulant role in maintaining hemostatic balance. However, the effect was smaller at follow-up, indicating that COCs downregulate this natural anticoagulant, thereby contributing to the prothrombotic state.

Earlier findings demonstrated that TG is higher in the absence of PS, as assessed by adding antibodies against PS [37,42,46]. In our previous study [54], we showed that the PS activity was lower in the follow-up samples, but the decrease in PS activity was quite small (7%). The present results indicate that the impact of PS is of great significance for TG, since we observed a considerable increase in ETP and Peak values, both at baseline and attenuated due to COCs at the follow-up. Interestingly, no overall correlation could be seen between PS activity and ETP, but the PS activity assay only detects an effect in combination with APC whereas PS also has an important role as cofactor for TFPI.

### 4.3 Impact of TFPI inhibition

The primary role of TFPI is to inhibit the initiation phase of coagulation. TFPI has an α and a β variant, of which TFPIβ is cell surface bound and therefore not present in the TG assay. TFPIα (simply called TFPI below) binds to and inhibits FXa, and this complex inactivates the TF:FVIIa complex, preventing further activation of FX. PS is an important cofactor for TFPI in these reactions, increasing the efficacy of TFPI on the direct inhibition of FXa and subsequently TF:FVIIa [35,[37], [38], [39], [40]].

To investigate the effect of TFPI, we assessed TG after addition of aTFPI (ie, by removing the effect of TFPI), and we observed significantly higher TG than in the control situation in the presence of TFPI, both at baseline and after 3 months of COC use, although TG was attenuated at follow-up. The Peak value in particular was increased, and Lag time was shortened in accordance with the fact that TFPI inhibits the initiation of the coagulation. On the other hand, ETP was almost the same as after addition of aPS, showing that the “power” in the system is comparable in the presence of aPS or aTFPI, respectively. The results clearly indicate that TFPI plays an important anticoagulant role in maintaining hemostatic balance, and it is downregulated by COCs.

Regarding previous studies on the impact of TFPI on TG, a similar trend as for PS has been described, and the effect was reportedly more pronounced than for PS [37,38,[40], [41], [42], [43], [44], [45], [46], [47]]. We have earlier [54] investigated the impact of COCs on TFPI levels, which were lowered, but the reduction in TFPI was relatively small, approximately 10%, whereas the present results indicate that the reduction of TFPI obviously has a great impact on TG.

We also assessed TG after adding both aTFPI and APC, enhancing the effect of the protein C:PS complex while removing the influence of TFPI. At baseline, we observed greater TG than in the control situation, along with slightly higher ETP and significantly higher Peak. Compared with addition APC, TG is substantially increased by addition of aTFPI. This underlines a very important effect of TFPI. At the follow-up, ETP and Peak were significantly higher, but the increase was relatively smaller than in the control situation. If we calculate a sensitivity ratio of APC in the situation without TFPI, ie, ETP (+APC and +aTFPI) / ETP(−APC and +aTFPI), this ratio only increases 18% from baseline to follow-up compared with an approximate doubling when TFPI is present. This shows that the effect of stimulating PS by APC can be substantially counteracted by inhibition of TFPI, clearly illustrating the profound importance of TFPI, and underscores that the effect is reduced after COC initiation, emphasizing that the effect on TFPI by COCs is substantial.

### 4.4 Clinical implications

TG assay is used to monitor and quantify all pro- and anticoagulant alterations in the blood, thereby assessing overall thrombogenicity. Tchaikovski et al. [28] proposed that a substantial portion of the increased thrombogenicity observed during COC use is attributable to reductions of the system inhibitors, specifically PS and TFPI. The findings of this study are in accordance with this view, indicating a substantial impact of these COC-induced alterations on overall TG.

Addition of aPS and aTFPI demonstrated the substantial influence of these anticoagulants, which was much more marked than measuring PS and TFPI activity directly. Therefore, it could potentially be a way to examine the effect of COCs in women prone to thrombosis as the reduction of these anticoagulants may be a pivotal part of the thrombogenicity of COCs.

A key strength of this study is the detailed assessment of TG under various conditions, providing insight into the procoagulant effects of COCs, and it was performed in the same individuals before and after start of COCs. To prevent activation of the contact system, all blood samples related to TG were drawn into Monovette tubes [68]. The main limitation of this study was the sample size, which is relatively small, limiting the generalizability of the findings.

We could have tested the effect of aPS and aTFPI in combination. However, assessing TG combining aPS and aTFPI has been done before and showed that reduction of PS activity in the absence of TFPI activity does not increase TG activity [35,37,46].

## 5 Conclusion

Our study shows that, even though the COC-induced decreases in PS activity and TFPI levels can be relatively small, they are of great importance to overall coagulation ability, as the impact on TG, after 3 months of COC treatment, is both striking and prominent.

## References

[1] United Nations, Department of Economic and Social Affairs, Population Division (2019).

[2] Morimont L., Haguet H., Dogné J.M., Gaspard U., Douxfils J. (2021). Combined oral contraceptives and venous thromboembolism: review and perspective to mitigate the risk. Front Endocrinol (Lausanne).

[3] Baratloo A., Safari S., Rouhipour A., Hashemi B., Rahmati F., Motamedi M. (2014). The risk of venous thromboembolism with different generation of oral contraceptives; a systematic review and meta-analysis. Emerg (Tehran).

[4] Lidegaard Ø., Løkkegaard E., Svendsen A.L., Agger C. (2009). Hormonal contraception and risk of venous thromboembolism: national follow-up study. BMJ.

[5] Lidegaard Ø., Nielsen L.H., Skovlund C.W., Skjeldestad F.E., Løkkegaard E. (2011). Risk of venous thromboembolism from use of oral contraceptives containing different progestogens and oestrogen doses: Danish cohort study, 2001-9. BMJ.

[6] Plu-Bureau G., Maitrot-Mantelet L., Hugon-Rodin J., Canonico M. (2013). Hormonal contraceptives and venous thromboembolism: an epidemiological update. Best Pract Res Clin Endocrinol Metab.

[7] Practice Committee of the American Society for Reproductive Medicine (2017). Combined hormonal contraception and the risk of venous thromboembolism: a guideline. Fertil Steril.

[8] van Vlijmen E.F., Brouwer J.L., Veeger N.J., Eskes T.K., de Graeff P.A., van der Meer J. (2007). Oral contraceptives and the absolute risk of venous thromboembolism in women with single or multiple thrombophilic defects: results from a retrospective family cohort study. Arch Intern Med.

[9] Allen R.H. (2024). https://www.uptodate.com/contents/combined-estrogen-progestin-oral-contraceptives-patient-selection-counseling-and-use.

[10] Shahnazi M., Farshbaf Khalili A., Ranjbar Kochaksaraei F., Asghari Jafarabadi M., Gaza Banoi K., Nahaee J. (2014). A comparison of second and third generations combined oral contraceptive pills’ effect on mood. Iran Red Crescent Med J.

[11] Stanczyk F.Z., Mathews B.W., Cortessis V.K. (2017). Does the type of progestin influence the production of clotting factors?. Contraception.

[12] van Hylckama Vlieg A., Helmerhorst F.M., Vandenbroucke J.P., Doggen C.J., Rosendaal F.R. (2009). The venous thrombotic risk of oral contraceptives, effects of oestrogen dose and progestogen type: results of the MEGA case-control study. BMJ.

[13] Douxfils J., Raskin L., Didembourg M., Donis N., Dogné J.M., Morimont L. (2024). Are natural estrogens used in contraception at lower risk of venous thromboembolism than synthetic ones? A systematic literature review and meta-analysis. Front Endocrinol (Lausanne).

[14] Foidart J.M., Gemzell-Danielsson K., Kubba A., Douxfils J., Creinin M.D., Gaspard U. (2023). The benefits of estetrol addition to drospirenone for contraception. AJOG Glob Rep.

[15] Fruzzetti F., Fidecicchi T., Montt Guevara M.M., Simoncini T. (2021). Estetrol: a new choice for contraception. J Clin Med.

[16] Lee A., Syed Y.Y. (2022). Estetrol/drospirenone: a review in oral contraception. Drugs.

[17] Gemzell-Danielsson K., Cagnacci A., Chabbert-Buffet N., Douxfils J., Foidart J.M., Kubba A. (2022). A novel estetrol-containing combined oral contraceptive: European expert panel review. Eur J Contracept Reprod Health Care.

[18] Bonnar J. (1987). Coagulation effects of oral contraception. Am J Obstet Gynecol.

[19] Mackie I.J., Piegsa K., Furs S.A., Johnson J., Bounds W., Machin S.J. (2001). Protein S levels are lower in women receiving desogestrel-containing combined oral contraceptives (COCs) than in women receiving levonorgestrel-containing COCs at steady state and on cross-over. Br J Haematol.

[20] Trenor C.C., Chung R.J., Michelson A.D., Neufeld E.J., Gordon C.M., Laufer M.R. (2011). Hormonal contraception and thrombotic risk: a multidisciplinary approach. Pediatrics.

[21] Holmegard H.N., Nordestgaard B.G., Schnohr P., Tybjaerg-Hansen A., Benn M. (2014). Endogenous sex hormones and risk of venous thromboembolism in women and men. J Thromb Haemost.

[22] Raps M., Helmerhorst F.M., Fleischer K., Dahm A.E., Rosendaal F.R., Rosing J. (2013). The effect of different hormonal contraceptives on plasma levels of free protein S and free TFPI. Thromb Haemost.

[23] Tchaikovski S.N., van Vliet H.A., Thomassen M.C., Bertina R.M., Rosendaal F.R., Sandset P.M. (2007). Effect of oral contraceptives on thrombin generation measured via calibrated automated thrombography. Thromb Haemost.

[24] Westhoff C.L., Pike M.C., Cremers S., Eisenberger A., Thomassen S., Rosing J. (2017). Endogenous thrombin potential changes during the first cycle of oral contraceptive use. Contraception.

[25] Mohamed A.B.O., Kelchtermans H., Konings J., van Daal J., Al Marzouki A., Harakeh S. (2018). The effects of oral contraceptive usage on thrombin generation and activated protein C resistance in Saudi women, with a possible impact of the body mass index. PLoS One.

[26] Curvers J., Thomassen M.C., Nicolaes G.A., Van Oerle R., Hamulyak K., Hemker H.C. (1999). Acquired APC resistance and oral contraceptives: differences between two functional tests. Br J Haematol.

[27] Hugon-Rodin J., Alhenc-Gelas M., Hemker H.C., Brailly-Tabard S., Guiochon-Mantel A., Plu-Bureau G. (2017). Sex hormone-binding globulin and thrombin generation in women using hormonal contraception. Biomarkers.

[28] Tchaikovski S.N., Rosing J. (2010). Mechanisms of estrogen-induced venous thromboembolism. Thromb Res.

[29] Sandset P.M. (2013). Mechanisms of hormonal therapy related thrombosis. Thromb Res.

[30] Gierula M., Ahnström J. (2020). Anticoagulant protein S—new insights on interactions and functions. J Thromb Haemost.

[31] Mahmoodi B.K., Brouwer J.L., Ten Kate M.K., Lijfering W.M., Veeger N.J., Mulder A.B. (2010). A prospective cohort study on the absolute risks of venous thromboembolism and predictive value of screening asymptomatic relatives of patients with hereditary deficiencies of protein S, protein C or antithrombin. J Thromb Haemost.

[32] Alshaikh N.A. (2022). Protein S: a central regulator of blood coagulation. Clin Lab.

[33] Majumder R., Nguyen T. (2021). Protein S: function, regulation, and clinical perspectives. Curr Opin Hematol.

[34] Alshehri F.S., Bashmeil A.A., Alamar I.A., Alouda S.K. (2024). The natural anticoagulant protein S; hemostatic functions and deficiency. Platelets.

[35] Castoldi E., Hackeng T.M. (2008). Regulation of coagulation by protein S. Curr Opin Hematol.

[36] Sedzro J.C., Adam F., Auditeau C., Bianchini E., De Carvalho A., Peyron I. (2022). Antithrombotic potential of a single-domain antibody enhancing the activated protein C-cofactor activity of protein S. J Thromb Haemost.

[37] Hackeng T.M., Seré K.M., Tans G., Rosing J. (2006). Protein S stimulates inhibition of the tissue factor pathway by tissue factor pathway inhibitor. Proc Natl Acad Sci U S A.

[38] Hackeng T.M., Rosing J. (2009). Protein S as cofactor for TFPI. Arterioscler Thromb Vasc Biol.

[39] Ahnström J., Petri A., Crawley J.T.B. (2024). Tissue factor pathway inhibitor – cofactor-dependent regulation of the initiation of coagulation. Curr Opin Hematol.

[40] Petri A., Sasikumar P., Folgado P.B., Jones D., Xu Y., Ahnström J. (2024). TFPIα anticoagulant function is highly dependent on protein S in vivo. Sci Adv.

[41] Andersson H.M., Arantes M.J., Crawley J.T., Luken B.M., Tran S., Dahlbäck B. (2010). Activated protein C cofactor function of protein S: a critical role for Asp95 in the EGF1-like domain. Blood.

[42] Seré K.M., Rosing J., Hackeng T.M. (2004). Inhibition of thrombin generation by protein S at low procoagulant stimuli: implications for maintenance of the hemostatic balance. Blood.

[43] Alshaikh N.A., Rosing J., Thomassen M.C.L.G.D., Castoldi E., Simioni P., Hackeng T.M. (2017). New functional assays to selectively quantify the activated protein C- and tissue factor pathway inhibitor-cofactor activities of protein S in plasma. J Thromb Haemost.

[44] Maurissen L.F., Castoldi E., Simioni P., Rosing J., Hackeng T.M. (2010). Thrombin generation-based assays to measure the activity of the TFPI-protein S pathway in plasma from normal and protein S-deficient individuals. J Thromb Haemost.

[45] Hackeng T.M., Maurissen L.F., Castoldi E., Rosing J. (2009). Regulation of TFPI function by protein S. J Thromb Haemost.

[46] Rosing J., Maurissen L.F., Tchaikovski S.N., Tans G., Hackeng T.M. (2008). Protein S is a cofactor for tissue factor pathway inhibitor. Thromb Res.

[47] Reglińska-Matveyev N., Andersson H.M., Rezende S.M., Dahlbäck B., Crawley J.T., Lane D.A. (2014). TFPI cofactor function of protein S: essential role of the protein S SHBG-like domain. Blood.

[48] Kristensen S.R., Nybo J., Pedersen S. (2022). Thrombin generation measured on ST Genesia, a new platform in the coagulation routine lab: assessment of analytical and between-subject variation. Res Pract Thromb Haemost.

[49] Glintborg D., Sidelmann J.J., Altinok M.L., Mumm H., Andersen M. (2015). Increased thrombin generation in women with polycystic ovary syndrome: a pilot study on the effect of metformin and oral contraceptives. Metabolism.

[50] Morimont L., Jost M., Gaspard U., Foidart J.M., Dogné J.M., Douxfils J. (2022). Low thrombin generation in users of a contraceptive containing estetrol and drospirenone. J Clin Endocrinol Metab.

[51] Zermatten M.G., Bertaggia Calderara D., Aliotta A., Alberio L. (2020). Thrombin generation in a woman with heterozygous factor V Leiden and combined oral contraceptives: a case report. Res Pract Thromb Haemost.

[52] Haverinen A.H., Luiro K.M., Szanto T., Kangasniemi M.H., Hiltunen L., Sainio S. (2022). Combined oral contraceptives containing estradiol valerate vs ethinylestradiol on coagulation: a randomized clinical trial. Acta Obstet Gynecol Scand.

[53] Zia A., Callaghan M.U., Callaghan J.H., Sawni A., Bartlett H., Backos A. (2015). Hypercoagulability in adolescent girls on oral contraceptives-global coagulation profile and estrogen receptor polymorphisms. Am J Hematol.

[54] Strandberg J., Gade I.L., Nybo J., Thomsen J.N.L., Kristensen S.R. (2025). The effect of combined oral contraceptives on thrombin generation assessed on ST Genesia– a paired clinical study. Thromb J.

[55] Lacroix R., Judicone C., Poncelet P., Robert S., Arnaud L., Sampol J. (2012). Impact of pre-analytical parameters on the measurement of circulating microparticles: towards standardization of protocol. J Thromb Haemost.

[56] Kristensen A.F., Kristensen S.R., Falkmer U., Münster A.B., Pedersen S. (2018). Analytical and between-subject variation of thrombin generation measured by calibrated automated thrombography on plasma samples. Scand J Clin Lab Invest.

[57] Tchaikovski S.N., Thomassen M.C.L.G.D., Stickeler E., Bremme K., Rosing J. (2021). Resistance to activated protein C and impaired TFPI activity in women with previous hormone-induced venous thromboembolism. Thromb Res.

[58] Kristensen S.R., Nybo J. (2023). A sensitive tissue factor activity assay determined by an optimized thrombin generation method. PLoS One.

[59] Rosing J., Tans G., Nicolaes G.A., Thomassen M.C., van Oerle R., van der Ploeg P.M. (1997). Oral contraceptives and venous thrombosis: different sensitivities to activated protein C in women using second- and third-generation oral contraceptives. Br J Haematol.

[60] Morimont L., Bouvy C., Delvigne A.S., Dogné J.M., Douxfils J. (2020). Proof of concept of a new scale for the harmonization and the standardization of the ETP-based APC resistance. J Thromb Haemost.

[61] Oral Contraceptive and Hemostasis Study Group (2003). The effects of seven monophasic oral contraceptive regimens on hemostatic variables: conclusions from a large randomized multicenter study. Contraception.

[62] Douxfils J., Klipping C., Duijkers I., Kinet V., Mawet M., Maillard C. (2020). Evaluation of the effect of a new oral contraceptive containing estetrol and drospirenone on hemostasis parameters. Contraception.

[63] de Bastos M., Stegeman B.H., Rosendaal F.R., Van Hylckama Vlieg A., Helmerhorst F.M., Stijnen T. (2014). Combined oral contraceptives: venous thrombosis. Cochrane Database Syst Rev.

[64] Marchetti M., Castoldi E., Spronk H.M., van Oerle R., Balducci D., Barbui T. (2008). Thrombin generation and activated protein C resistance in patients with essential thrombocythemia and polycythemia vera. Blood.

[65] Høibraaten E., Mowinckel M.C., de Ronde H., Bertina R.M., Sandset P.M. (2001). Hormone replacement therapy and acquired resistance to activated protein C: results of a randomized, double-blind, placebo-controlled trial. Br J Haematol.

[66] van Vliet H.A., Rodrigues S.P., Snieders M.N., van der Meer F.J., Frolich M., Rosendaal F.R. (2008). Sensitivity to activated protein C during the menstrual cycle in women with and without factor V_Leiden_. Thromb Res.

[67] Segers O., Simioni P., Tormene D., Bulato C., Gavasso S., Rosing J. (2012). Genetic modulation of the FV_Leiden_/normal FV ratio and risk of venous thrombosis in factor V Leiden heterozygotes. J Thromb Haemost.

[68] Kristensen S.R., Gram J.B., Nybo J., Sidelmann J.J., Palarasah Y. (2023). Estimation of the preanalytical activation of the contact system in coagulation tubes. Thromb Res.

